# Near-Infrared Spectroscopy as a Novel Non-Invasive Tool to Assess Spiny Lobster Nutritional Condition

**DOI:** 10.1371/journal.pone.0159671

**Published:** 2016-07-21

**Authors:** Cedric J. Simon, Thomas Rodemann, Chris G. Carter

**Affiliations:** 1 Fisheries and Aquaculture Centre, Institute for Marine and Antarctic Studies, University of Tasmania, Nubeena Crescent, Taroona, TAS 7053, Australia; 2 Central Science Laboratory, University of Tasmania, Hobart, 7001, Australia; CNR, ITALY

## Abstract

Rapid non-invasive monitoring of spiny lobster nutritional condition has considerable application in the established fishery, live market and prospective aquaculture. The aim of this research was to test the feasibility of near-infrared spectroscopy (NIRS) as a novel non-invasive tool to assess the nutritional condition of three lobster species. Lobster (*n* = 92) abdominal muscle dry matter (AM_DM_) and carbon content (AM_C_) correlated significantly with indices of nutritional condition including hepatopancreas dry matter (HP_DM_; r_ho_ = 0.83, 0.78), total lipid content (HP_TL_; r_ho_ = 0.85, 0.87) and haemolymph total protein (TP; r_ho_ = 0.89, 0.87 respectively). Abdominal muscle nitrogen content (AM_N_) was a poor correlate of nutritional condition. Models based on FT-NIR scanning of whole lobster tails successfully predicted AM_DM_, AM_N_ and AM_C_ (RMSECV = 1.41%, 0.35% and 0.91%; R^2^ = 0.75, 0.65, 0.77, respectively), and to a lower accuracy HP_DM_, HP_TL_ and TP (RMSECV = 6.22%, 8.37%, 18.4 g l^-1^; R^2^ = 0.51, 0.70, 0.83, respectively). NIRS was applied successfully to assess the condition of spiny lobsters non-invasively. This pilot study paves the way for the development of crustacean condition models using portable non-invasive devices in the laboratory or in the field.

## Introduction

Spiny lobsters support some of the most valuable commercial wild fisheries worldwide. Demand for spiny lobsters is increasing at a time when many wild stocks are fully exploited and some major fisheries are declining due to overfishing, poor recruitment, and possibly climate change [1]. Particularly, changes in water temperature and food availability can affect the growth and nutritional condition of spiny lobsters [2], and environmental conditions have important consequences on recruitment and spiny lobster fisheries production [1]. Spiny lobsters are marketed principally as a live product and current industry best practices focus on minimising physical and environmental stressors in the context of improving the health status and survival during live transport [1]. While key market traits influencing price include shell coloration, size and body shape, there has been little attention given to the variability in meat quality [3]. A better understanding of the factors influencing variability in meat quality could assist the industry in improving post-harvest processing, management of fattening, and control of when to release for sale [3,4,5]. For the consumer, a lobster of high tail meat yield, with edible tissues rich in protein and omega-3 lipid, as well as the expected taste and texture should be seen as highly favourable attributes.

A practical, rapid and non-invasive technique to analyse lobster nutritional condition has considerable potential to assist with the management of wild stocks [2,3,6], improving post-harvest survival and quality [1], and in managing broodstock and juvenile lobsters as an emergent lobster aquaculture industry expands to meet the growing demand [7]. Several indices of nutritional condition that require euthanizing the lobsters have been based on the biochemical changes in hepatopancreas (HP) such as its dry matter (HP_DM_) or total lipid content (HP_TL_) [2,8,9,10]. The hepatopancreas is typically high in lipid and responds rapidly to changes in physiological and environmental parameters such as food deprivation, moulting and reproduction [9]. In contrast, changes in abdominal muscle tissue composition has received less attention as the current understanding is membrane-bound phospholipids do not respond readily to short-term changes in physiological and environmental parameters [2]. Nevertheless, abdominal muscle composition, including water content, percent carbon and nitrogen, changes are evident after severe physiological stress associated with annual breeding migration and starvation in *Panulirus ornatus* [11]. The slow changes in muscle tissue are useful in understanding the longer term dietary changes experienced by the animal and are more important from a commercial and consumer perspective as they relate directly to edible meat yield and quality [3]. Some markets do however also value the hepatopancreas (also called tomalley in North America), which can be eaten alone or added to sauces, despite the potential health risk associated with heavy metal contamination [12]. There are very limited useful non-invasive condition indices that have been developed for crustaceans that would be suitable when immediate answers are required in the field or when a large number of animals need to be sampled. The absence of quantifiable changes in external dimensions during the periods of sub-satiation feeding or starvation makes traditional morphometric condition factors poorly applicable to lobsters [13]. Limited wet weight change occurs within intermoult because the metabolised substrates within internal soft tissues are replaced by an equivalent amount of water during starvation [14]. While the ratio of whole body wet weight to carapace length has been used in many crustacean studies, the strength of its relationship to condition differs with species, and it is generally slow to respond to periods of starvation in lobster [13]. Haemolymph total protein concentration, and its approximation by refractometry, has been used as the main non-invasive index of condition in crustaceans [13,15,16]. Haemolymph protein concentration however varies with a range of environmental stressors as well as the moult cycle, reproductive cycle and nutrition, so it is important to take all these factors into account when considering its use as a predictor of vitality prior to live shipment or nutritional condition of lobsters [6,13,15,17]. Condition assessment in spiny lobsters based solely on protein concentration is also particularly problematic because of the long intermoult stage and difficulties in distinguishing between the beginning and end of the intermoult [6,15,18].

Near-infrared reflectance spectroscopy (NIRS) is a non-invasive technique that has been increasingly used for evaluation of seafood quality [19]. NIRS can provide rapidly extensive information on fish quality and has been successfully used to predict chemical composition, in terms of fat, protein and moisture, microbiological and sensory attributes of a range of seafood products including live fish, whole fish fillets, fresh minced samples, freeze-dried samples and fish sauce [19]. The NIR spectra can be recorded in reflection, transmission and transflection modes over a waveband between 700 and 2,500 nm and provide complex structural information related to the fundamental vibration behaviour of molecular bonds such as C-H, O-H, and N-H in the mid-infrared (MIR) [19]. The lower degree of absorbance of NIR, and therefore higher degree of reflected light, compared to MIR, is due to the reduced excitation state of harmonics and combination vibration. This makes NIRS a practical non-invasive portable tool as it can be integrated into reflectance optic probes to measure unprocessed samples that can be a few mm thick [20]. Prediction of chemical content by non-invasive measurements of whole fish is more challenging than processed tissues because the skin absorbs and reflect in the NIR region thus making it difficult to obtain representative measurements from the interior of the fish muscle, yet promising results have been achieved in measuring total lipid in whole and live salmon [19, 21,22]. NIRS has been applied successfully to predict chemical composition of only a few commercial invertebrates, including shucked and homogenised oysters [23] and live abalone [24]. NIRS was shown to be a useful detection tool for adulteration in crab [25] but could not be used in predicting meat attributes of whole and minced cooked shrimp [26]. To our knowledge, NIRS has not been applied to any whole unprocessed crustacean species, including spiny lobsters.

The aims of this study were to validate the use of abdominal muscle composition as a useful indicator of nutritional condition in different spiny lobster species and sizes and evaluate NIRS as a potential non-invasive tool for measuring nutritional condition.

## Material and Methods

### Experimental lobsters, feeding and rearing system

Three commercial spiny lobster species with varying individual sizes were used in this study to cover a broad range of spiny lobster biological attributes. Cultured eastern spiny lobster juveniles (17–55 g; *n* = 35), *Sagmariasus verreauxi*, wild-caught southern spiny lobster juveniles and adults (107–1484 g; *n* = 33), *Jasus edwardsii*, and cultured tropical spiny lobster juveniles (104–377 g; *n* = 24), *Panulirus ornatus*, were used in this study and maintained according to previously published methods [27,28,29]. Cultured lobsters were produced from domesticated broodstock in hatchery at the Institute for Marine and Antarctic Studies (IMAS). Wild-caught lobsters were harvested on a commercial fishing vessel under an IMAS research permit between February-April 2012. Lobsters (*n* = 92) were held in laboratory-controlled states of feeding or food deprivation for up to 6 weeks to achieve a large range in nutritional condition. Fed lobsters received fresh half-shell mussels, *Mytilus edulis*, daily [28]. Lobsters were held individually in flow-through tanks fitted with airstones and supplied with filtered ozonated seawater with temperatures of 18°C for *J*. *edwardsii*, 21°C for *S*. *verreauxi*, and 26°C for *P*. *ornatus*. Artificial shelters made of oyster mesh were placed in tanks to provide lobsters with a substrate and shelter to minimize stress [28]. Tanks were cleaned every 14 days. Photoperiod was 12:12 h light:dark and the water quality maintained within established levels for these species [27,28,29].

### Haemolymph total protein

Haemolymph was collected aseptically from all live lobsters. Around 1 ml was withdrawn from the base of the 5^th^ leg within 1 min of emersion using a 3 ml syringe and 26 G Terumo needles kept on crushed ice. The haemolymph was centrifuged at 3,000 × *g* for 3 min, the plasma removed into a clean Eppendorf tube, frozen at −80°C and freeze-dried. The plasma samples were reconstituted with a known volume of deionized water calculated from the weight difference after drying, and analysed for total protein (TP; in mg ml^-1^) on a Cobas c501 automated biochemistry analyzer (Roche Diagnostics Corporation, Indianapolis, IN, USA).

### Hepatopancreas and abdominal muscle composition

Lobsters (*n* = 92) were destructively sampled immediately after taking the haemolymph sample by bringing them to a chill-coma on ice. Lobsters were weighed (extracted haemolymph volume accounted for) and the hepatopancreas (HP) dissected out and weighed (± 0.01 g) into duplicate samples. The entire abdomen was severed from the cephalothorax and kept whole frozen at −20°C for less than three months until sampled for NIRS. The abdominal muscle (AM) was then dissected out and weighed (± 0.01 g) into triplicate samples. Dry matter content of the tissues (HP and AM) was analysed by weight change following freeze-drying to a constant weight as follows:

HPDM (%) = HP tissue freeze−dried weight (HPDW) / HP tissue wet weight (HPWW) × 100

AMDM (%) = AM tissue freeze−dried weight (AMDW) / AM tissue wet weight (AMWW) × 100

AM samples were ground using a mortar and pestle and analysed for total nitrogen (AM_N_) and total carbon (AM_C_) by elemental analysis using a Thermo Finnigan EA 1112 Series Flash Elemental Analyser (Central Science Laboratory, University of Tasmania). Total lipid content of HP (HP_TL_) was determined using chloroform: methanol: water (1: 1: 0.9) [30] and expressed in % of dry matter.

### Near-infrared reflectance spectroscopy (NIRS)

The ventral side of whole frozen-thawed (1–4°C) lobster abdomens was chosen for non-invasive NIRS sampling as it features a thin flexible chitinous membrane in between segments. The large flexor muscle lies just beneath this chitinous membrane allowing for a suitable *in-vivo* NIRS light penetration and reflection from the internal soft tissue of the lobster. We attempted to predict both the lobster abdominal muscle composition as well as the hepatopancreas composition, even if the latter was not the targeted tissue during NIRS sampling. The ability to predict haemolymph protein was also investigated. The ventral part of the abdomen was pat dried and pleopods moved to the side in preparation for NIRS measurements. A total of six areas located on both sides of the abdominal aorta at the second, third and fourth segments were scanned using diffuse reflectance fibre optic probes with a sampling area of 2mm or alternatively 3.5mm diameter, and averaged. The NIR spectra were recorded on a Bruker MPA FT-NIR spectrometer using an 8 cm^-1^ resolution between 12500 and 4000 cm^-1^ with 32 scans for the background and each sampling area. Different spectral regions of interest and transformations were applied for the partial least square regression of AM_DM_, AM_N_, AM_C_, HP_DM_, HP_TL_, and TP. The separate regression models were developed using 91 samples with a random cross-validation (20 segments).

### Statistical analyses

Correlations between haemolymph protein, hepatopancreas composition and abdomen muscle composition were tested statistically using Spearman’s rank test (*P*<0.05). All analyses were performed using SPSS 21.0. Best-fit quadratic regressions (second degree polynomial) were tested statistically in Sigmaplot 21.0.

The statistics calculated for assessing the robustness of the calibration models included the number of principal components (PCs), correlation coefficient between predicted and measured composition (R^2^), and the standard error of cross-validation (RMSECV). RMSECV is the standard deviation of differences of the residuals between the NIRS and chemically determined concentrations and has been reported to be the best estimate for the prediction capability of calibrations [5]. The best models were selected based on the lowest RMSECV and the highest R^2^ of the cross-validations. The residual predictive deviation (RPD) calculated as standard deviation of the measured composition (SD)/RMSECV was used to evaluate the performance of the calibrations [31]. Partial least square regression (PLSR) modelling was performed in Unscrambler^®^ X.

## Results

### Nutritional condition indices

**Fig 1:**
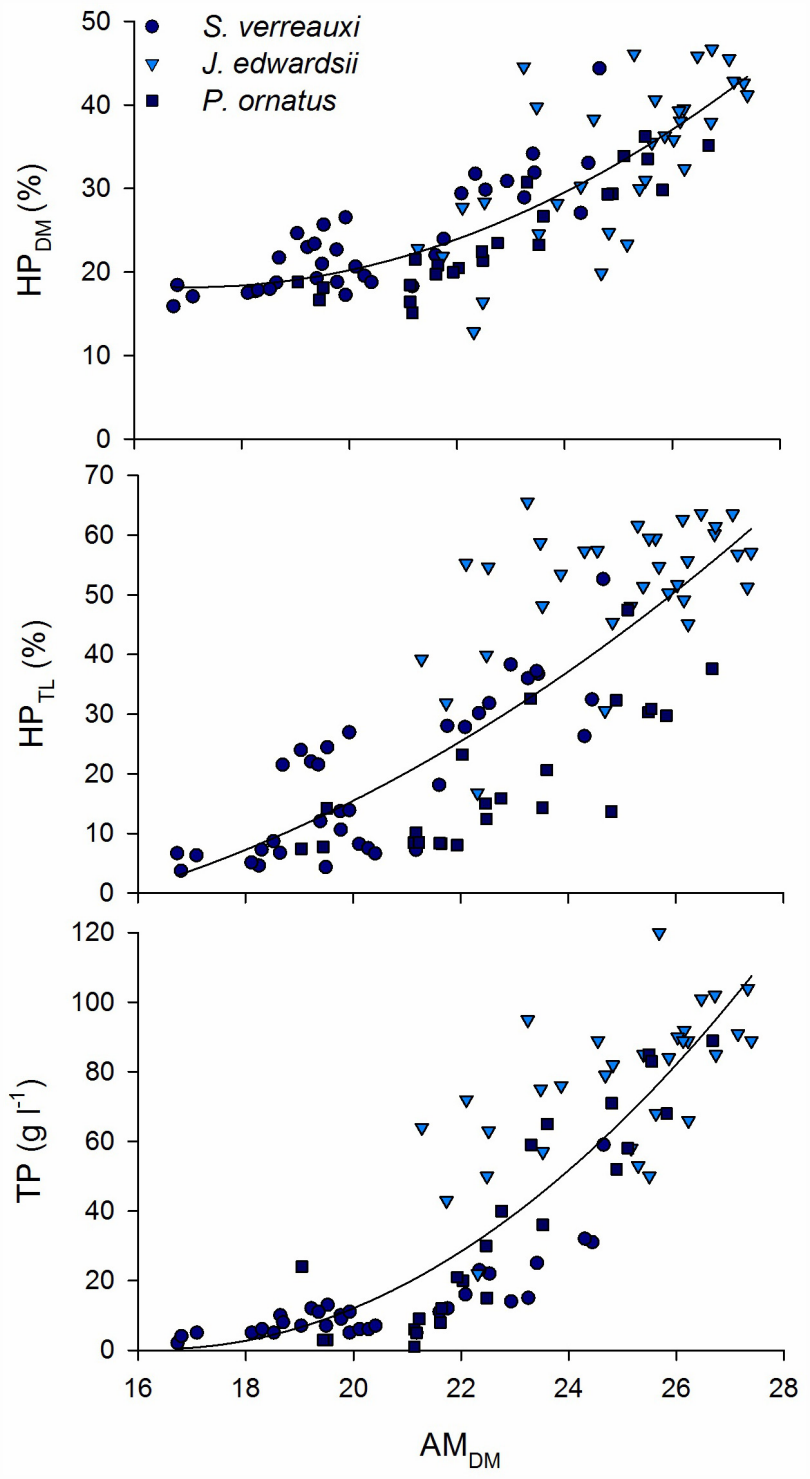
Relationship between abdominal muscle dry matter content (AM_DM_) and hepatopancreas dry matter content (HP_DM_), hepatopancreas total lipid content (HP_TL_), and haemolymph total protein (TP) for all three species, *Sagmariasus verreauxi*, *Jasus edwardsii*, and *Panilurus ornatus*.

**Fig 2:**
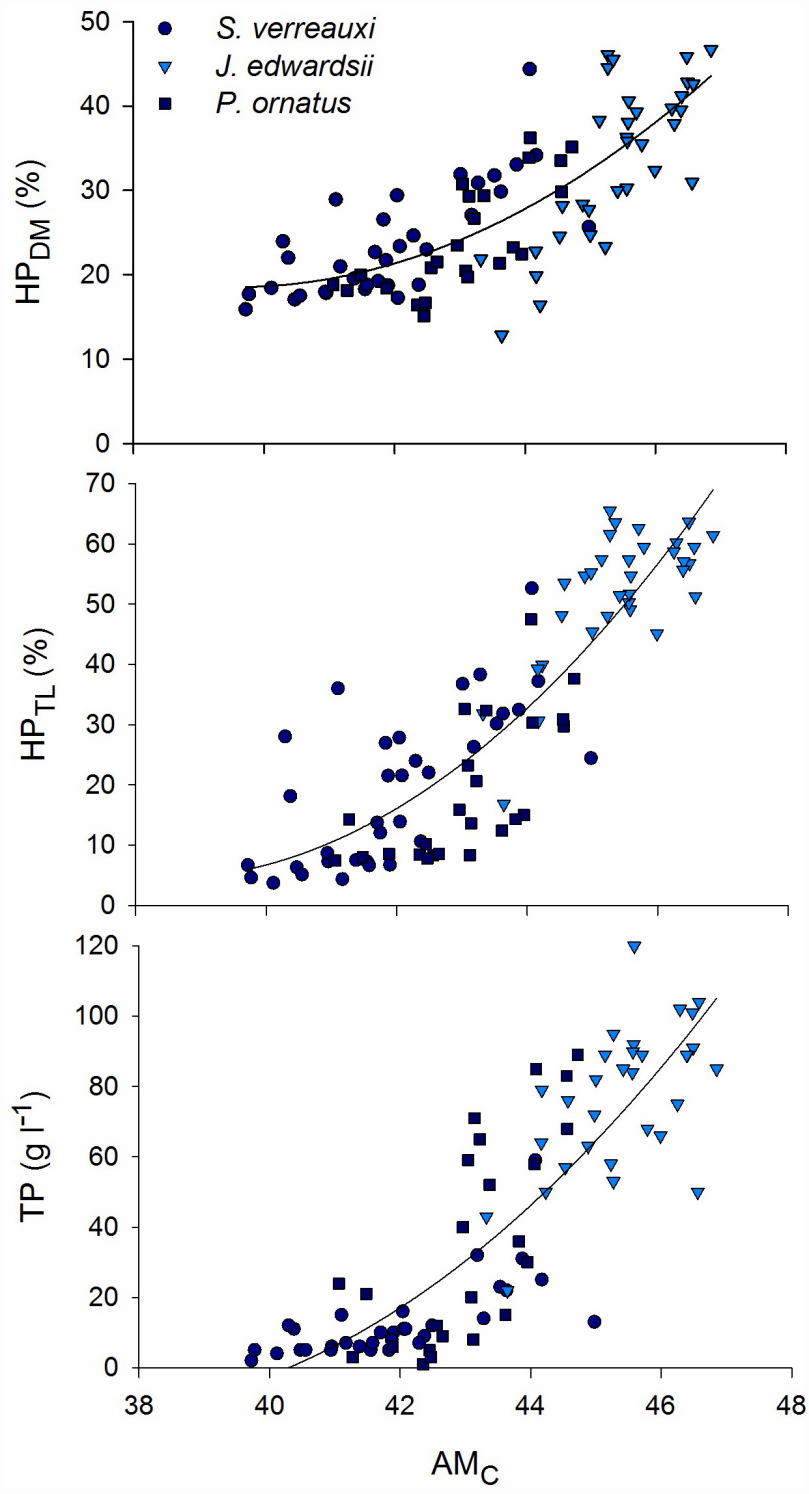
Relationship between abdominal muscle nitrogen content (AM_C_) and hepatopancreas dry matter content (HP_DM_), hepatopancreas total lipid content (HP_TL_), and haemolymph total protein (TP) for all three species, *Sagmariasus verreauxi*, *Jasus edwardsii*, and *Panilurus ornatus*.

**Fig 3:**
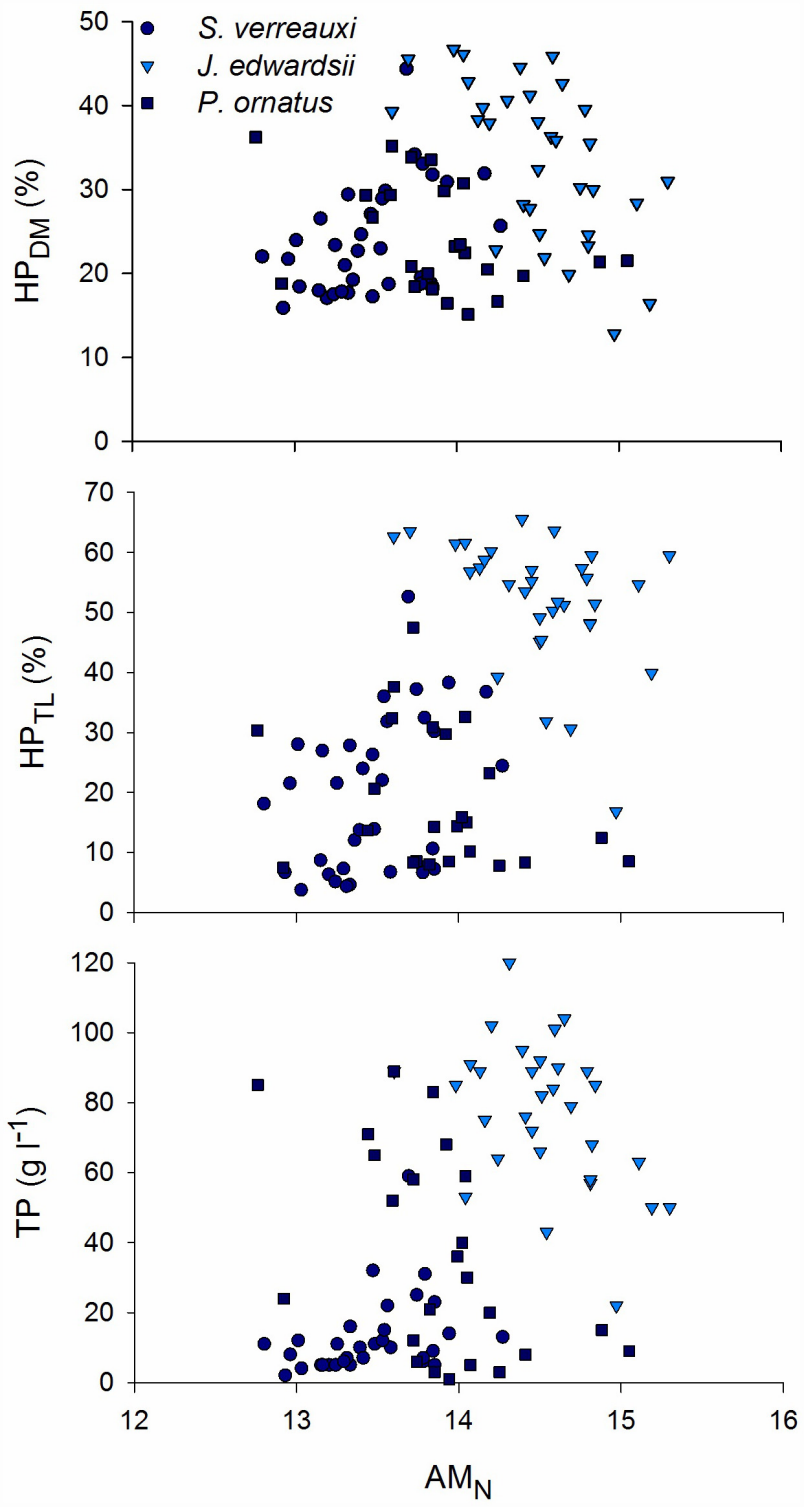
Relationship between abdominal muscle carbon content (AM_N_) and hepatopancreas dry matter content (HP_DM_), hepatopancreas total lipid content (HP_TL_), and haemolymph total protein (TP) for all three species, *Sagmariasus verreauxi*, *Jasus edwardsii*, and *Panilurus ornatus*.

The combined dataset provided a large variation in haemolymph total protein (%CV = 83.1), hepatopancreas (HP) dry matter (%CV = 32.3) and total lipid (%CV = 64.3) (Table 1). Abdominal muscle (AM) composition was less variable (%CV ranged 4.3–12.4) (Table 1). The three species of spiny lobsters were sampled within different weight/size classes but showed overlapping distributions in terms of HP and AM composition as well as TP (Table 1). AM_DM_ (Fig 1) and AM_C_ (Fig 2) correlated significantly to HP_DM_, HP_TL_ and TP for all three species groups separately and combined (Table 2). The strongest correlations were found between AM_DM_ and haemolymph TP (*rho* = 0.89), and between AM_C_ and HP_TL_ (*rho* = 0.87). AM_N_ was found to correlate poorly to HP composition and TP (Fig 3; Table 2). The relationships between abdominal muscle and hepatopancreas composition, as well as abdominal muscle composition and TP, best-fitted second degree polynomials rather than being linear relationships (Figs 1 and 2). R^2^ of regression lines ranged 0.63–0.77 (Table 2). There was limited further decline in HP_DM_, HP_TL_ and TP as AM_DM_ decreased below 21% and AM_C_ decreased below 43% (Figs 1 and 2).

**Table 1:** Summary of body weight, abdominal muscle and hepatopancreas composition, and hemolymph total protein of the three spiny lobster species used in this study and combined.

Species	Parameter^a^	Min	Max	Mean ± S.D.	% CV
*S*.*verreauxi* (n = 35)	Body weight (g)	16.8	54.8	39.0 ± 9.9	25.3
	AM_DM_ (%)	16.7	24.6	20.4 ± 2.2	10.8
	AM_N_ (%)	12.8	14.3	13.5 ± 0.4	2.6
	AM_C_ (%)	39.7	45.0	41.9 ± 1.3	3.2
	HP_DM_ (%)	15.9	44.4	23.7 ± 6.4	27.2
	HP_TL_ (%)	3.7	52.6	19.1 ± 12.8	67.0
	TP (g l^-1^)	2.0	59.0	12.7 ± 11.3	88.6
*J*. *edwardsii* (n = 33)	Body weight (g)	112.0	1455.0	474.3 ± 287.1	60.5
	AM_DM_ (%)	21.3	27.4	24.9 ± 1.8	7.1
	AM_N_ (%)	13.6	15.3	14.5 ± 0.4	2.7
	AM_C_ (%)	43.3	46.9	45.4 ± 0.9	2.0
	HP_DM_ (%)	12.9	46.7	33.7 ± 9.3	27.6
	HP_TL_ (%)	16.9	65.5	52.1 ± 10.7	20.5
	TP (g l^-1^)	22.0	120.0	76.9 ± 20.9	27.1
*P*. *ornatus* (n = 24)	Body weight (g)	104.0	377.0	211.9 ± 79.2	37.4
	AM_DM_ (%)	19.0	26.7	22.8 ± 2.1	9.4
	AM_N_ (%)	12.8	15.0	13.9 ± 0.5	3.6
	AM_C_ (%)	41.1	44.7	43.1 ± 1.0	2.4
	HP_DM_ (%)	15.2	36.2	24.2 ± 6.5	26.6
	HP_TL_ (%)	7.5	47.5	18.6 ± 11.6	62.4
	TP (g l^-1^)	1.0	89.0	36.0 ± 29.7	82.5
All (*n* = 92)	Body weight (g)	16.8	1455.0	240.2 ± 257.4	107.1
	AM_DM_ (%)	16.7	27.4	22.7 ± 2.8	12.4
	AM_N_ (%)	12.8	15.3	14.0 ± 0.6	4.3
	AM_C_ (%)	39.7	46.9	43.5 ± 1.9	4.3
	HP_DM_ (%)	12.9	46.7	27.4 ± 8.9	32.3
	HP_TL_ (%)	3.7	65.5	30.8 ± 19.8	64.3
	TP (g l^-1^)	1.0	120.0	41.6 ± 34.6	83.1

^a^AM_DM_: abdominal muscle dry matter content;

AM_N_: abdominal muscle nitrogen content; AM_C_: abdominal muscle carbon content; HP_DM_: hepatopancreas dry matter content; HP_TL_: hepatopancreas total lipid content; TP: haemolymph total protein.

**Table 2:** Significant Spearman correlations coefficient (*P*<0.05) between abdominal muscle composition and other nutritional indices for each species and all lobsters combined. The equation and R^2^ of best-fit second degree polynomials are provided for AM_DM_ and AM_C_.

		Spearman correlation coefficients (P<0.05)				Regressions (Figs 1 and 2)		
Parameters^a^		*S*.*verreauxi*	*J*. *edwardsii*	*P*. *ornatus*	All	R^2^	Stat. (F, P-value)	Equation (y_0_, a, b) (y = y_o_ + ax +bx^2^)
	**HP**_**DM**_	0.80	0.69	0.93	0.83	0.68	96.1, <0.001	86.9, −8.1, 0.24
**AM**_**DM**_	**HP**_**TL**_	0.78	0.40	0.83	0.81	0.64	78.8, P<0.001	11.6, −4.2, 0.2
	**TP**	0.81	0.65	0.91	0.89	0.77	145.4, <0.001	234.9, −28.7, 0.2
	**HP**_**DM**_	0.45	−0.59	NA	0.27	----------------- NA ----------------		
**AM**_**N**_	**HP**_**TL**_	0.40	−0.47	NA	0.53	----------------- NA ----------------		
	**TP**	0.48	−0.41	−0.54	0.51	----------------- NA ----------------		
	**HP**_**DM**_	0.74	0.74	0.83	0.78	0.63	73.7, <0.001	756.8, −37.3, 0.5
**AM**_**C**_	**HP**_**TL**_	0.72	0.57	0.75	0.87	0.77	150.6, P<0.001	1384.8, −69.8, 0.9
	**TP**	0.76	0.57	0.76	0.87	0.74	120.8, <0.001	1713.2, −92.9, 1.3

^a^AM_DM_: abdominal muscle dry matter content;

AM_N_: abdominal muscle nitrogen content; AM_C_: abdominal muscle carbon content; HP_DM_: hepatopancreas dry matter content; HP_TL_: hepatopancreas total lipid content; TP: haemolymph total protein.

### Predicting nutritional condition with NIRS

**Fig 4:**
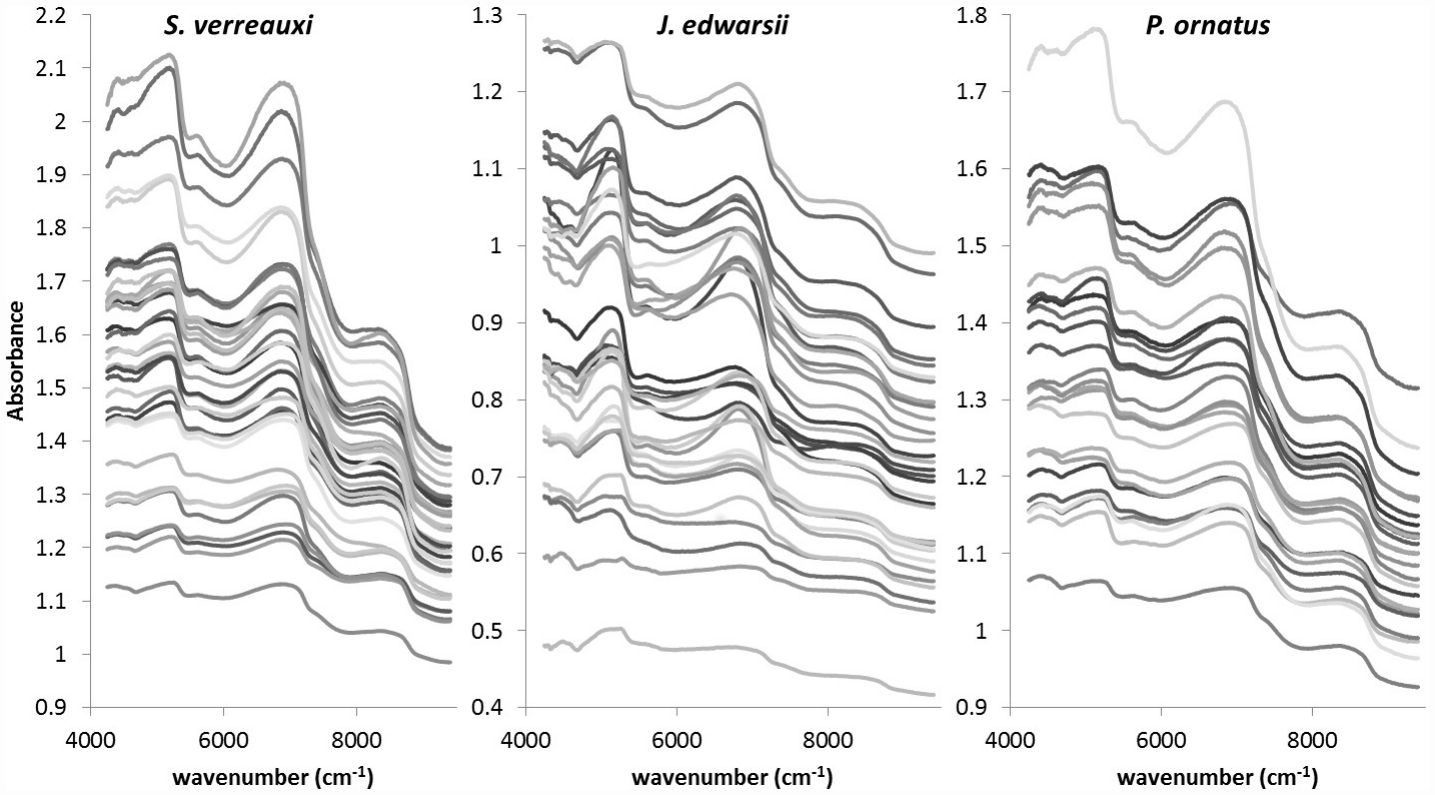
FT-NIR spectra of three spiny lobster species, *Sagmariasus verreauxi*, *Jasus edwardsii*, and *Panilurus ornatus*.

**Fig 5:**
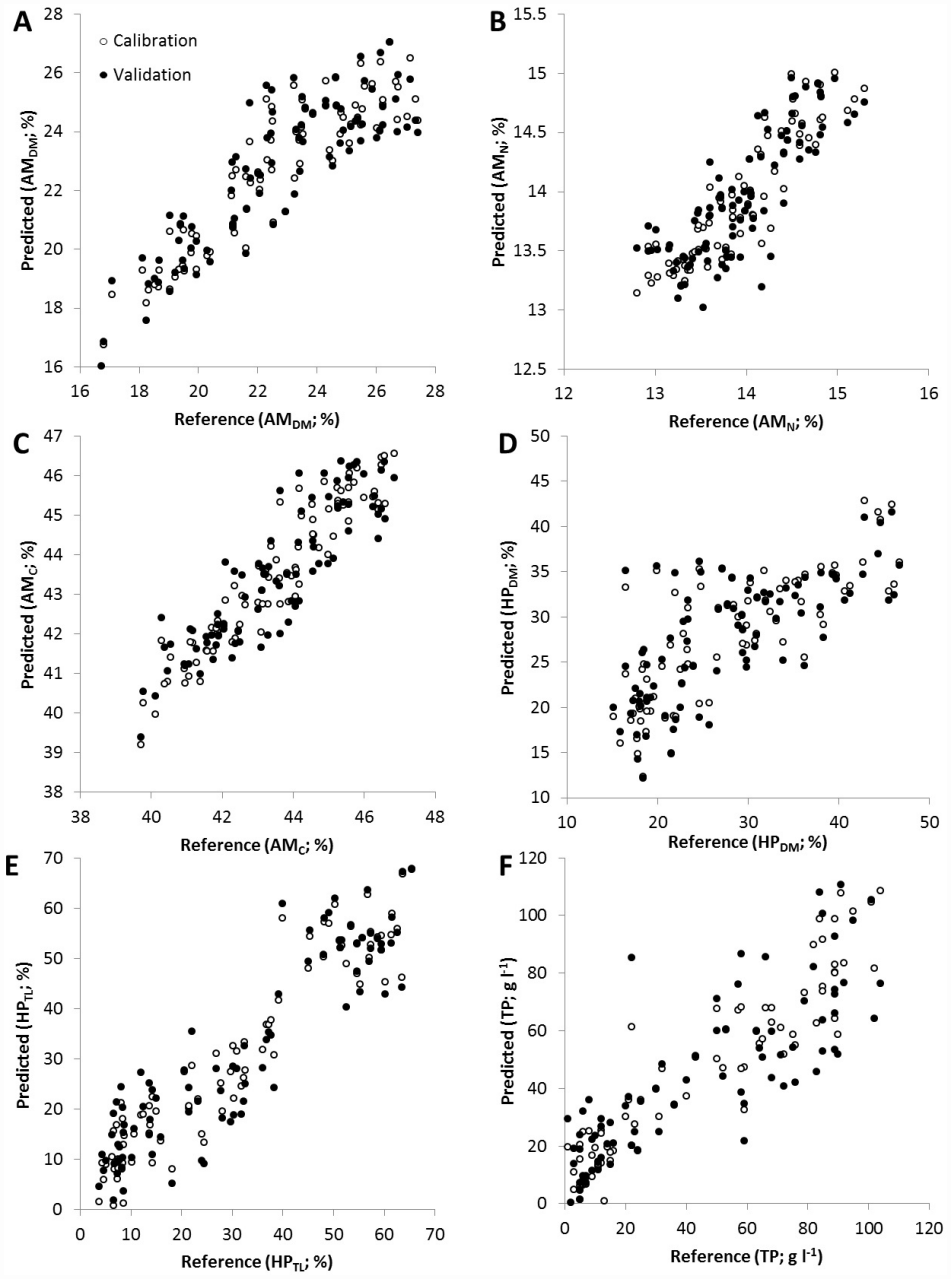
NIRS calibration and validation plots versus measured abdominal muscle A) dry matter, B) carbon, C) nitrogen, D) hepatopancreas dry matter, E) hepatopancreas total lipid content, and F) haemolymph total protein, for all lobster species combined.

The average spectra of whole lobster tails, expressed in wavenumber, are shown in Fig 4. Broad peaks were observed between 8800–8600 cm^-1^ (1130–1160 nm), 7400–6800 cm^-1^ (1350–1470 nm) and 5400–5100 cm^-1^ (1850–1950 nm) (Fig 4). Calibration models were improved by excluding spectral information in the range of 12500–9400 cm^-1^ and 4240–4000 cm^-1^. Only two regions between 9404–7498 cm^-1^ (1063–1334 nm) and 5450–4247 cm^-1^ (1834–2354 nm) contained useful information to model all our parameters of interest. The raw spectra was used for AM_DM_, HP_DM_ and HP_TL_ while spectral pre-treatments were applied for AM_N_ (1^st^ derivative), AM_C_ (baseline offset using linear corrections) and TP (SNV correction). Calibration models used 7 PCs for AM_DM_ and HP_TL_, 11for AM_N_, HP_DM_ and TP, and 12 for AM_C_ (Table 3). The correlation coefficient (R^2^) between actual (laboratory measured composition on freeze-dried samples) and NIRS predicted values from cross-validation ranged from 0.51 for HP_DM_ to 0.83 for HP_TL_ (Table 3; Fig 5). The average error of prediction (RMSECV) was 1.41% for AM_DM_, 0.35% for AM_N_, 0.91% for AM_C_, 6.22% for HP_DM_, 8.37% for HP_TL_ and 18.43 g l^-1^ for TP. RPD values for the models were between 1.4 and 2.4, with only AM_C_ and HP_TL_ models with RPD >2 (Table 3).

**Table 3:** Non-invasive prediction of lobster abdominal muscle and hepatopancreas composition as well as hemolymph total protein by NIRS. A total of 91 whole lobster tails were scanned using a FT-NIR instrument to develop the models. The model prediction is based on a random cross-validation with 20 segments.

Fig 5	Parameter^a^	Number of PCs	Correlation coefficient (R^2^)	RMSECV	RPD	Spectral data used	Wave number (cm-1)	wavelength (nm)
A	AM_DM_	7	0.75	1.41	1.9	Raw	9404–7498	1063–1334
B	AM_N_	11	0.65	0.35	1.7	1^st^ Derivative	9404–6098 & 5450–4247	1063–1640 & 1834–2354
C	AM_C_	12	0.77	0.91	2.1	Baseline offset using linear correction	9404–7498 & 5450–4598	1063–1334 & 1834–2175
D	HP_DM_	11	0.51	6.22	1.4	Raw	9404–7749	1063–1290
E	HP_TL_	7	0.83	8.37	2.4	Raw	9404–7498	1063–1334
F	TP	11	0.70	18.43	1.8	SNV correction	9404–7498 & 4848–4247	1063–1334 & 1710–2354

^a^ AM_DM_: abdominal muscle dry matter content;

AM_N_: abdominal muscle nitrogen content; AM_C_: abdominal muscle carbon content; HP_DM_: hepatopancreas dry matter content; HP_TL_: hepatopancreas total lipid content; TP: haemolymph total protein.

## Discussion

### Nutritional condition indices for spiny lobsters

Abdominal muscle dry matter and carbon content correlated to hepatopancreas dry matter and total lipid content as well as haemolymph protein for the three species tested in this study. The relationships were best described by second degree polynomial rather than a linear relationship. There was an indication that changes in hepatopancreas dry matter, total lipid and haemolymph protein indices were limited as abdominal dry matter and carbon content further reduced below 21% and 43% respectively. This trend was driven mainly by the smallest unfed individuals of the *S*. *verreauxi* species. The hepatopancreas of decapod crustaceans is primarily involved in the storage of lipid reserves [32] and is therefore typically the storage organ monitored to assess nutritional condition as it responds rapidly to food deprivation and moulting [2]. The dry matter and total lipid content of the hepatopancreas of adult male spiny lobster *Jasus lalandii* varied in accordance to the moult cycle through the year. Lowest dry matter content (17–28%) and total lipid (8–20% on dry weight basis) occurred at postmoult while highest dry matter (35–40%) and total lipid content (40–50%) occurred in late intermoult and early premoult [2]. In contrast, abdominal tail muscle dry matter content (22–26%), protein (84–94%) and total lipid content (0.2–2%) did not show a consistent variation or trend on a monthly or moult-cycle basis [2]. In our study, we managed feeding regimes to achieve a greater range in hepatopancreas dry matter (13–47%) and total lipid content (4–66%) for the entire dataset. This was also the case for abdominal muscle dry matter content which reached lowest level in unfed *S*. *verreauxi* juveniles (17%) and highest levels in fed *J*. *edwardsii* adults (27%). Starvation and satiation feeding for up to 6 weeks would have created the greater variation in nutritional condition observed in this study. Starvation causes a decrease in dry matter content of both the abdominal muscle and hepatopancreas in crustacean [33], including spiny lobsters such as *Panulirus cygnus* [14] and *J*. *edwardsii* [9]. In adult *P*. *ornatus*, the hepatopancreas is a more sensitive indicator of physiological stress than the muscle tissue but both were used for energy during migration (natural low feeding) and starvation [11]. Of the two tissues, the abdominal muscle is by far the most important energy reserve, with most of the energy stored as protein compared to total lipid in the hepatopancreas. Therefore, even a small noticeable decrease in abdominal muscle reserves can represent a large amount of absolute energy use in adult *P*. *ornatus* [11] and *S*. *verreauxi* [18]. The abdominal muscle protein energy reserve represented between 74 and 90% of the total energy reserve in juvenile lobsters, and as much as 40% can be used over 4 weeks of starvation [18]. The sequence of energy reserves use appear similar across spiny lobster species and size, with the lobsters drawing mainly on the energy reserves of the hepatopancreas first, even though the abdominal muscle is being used concurrently, then followed by greater reliance on abdominal muscle protein [11,18].

In our study, abdominal muscle carbon content correlated well to hepatopancreas total lipid whereas nitrogen (i.e., mainly protein) content did not. A similar decrease in abdominal muscle carbon, but not nitrogen, on a dry weight basis, occurs during natural loss of nutritional condition in *P*. *ornatus* [11]. As protein would contain both carbon and nitrogen, it is likely the carbon loss (AM_C_ ranged 39.7–46.9%) originated mainly from differential use of non-protein energy sources, which include abdominal muscle total lipid and carbohydrates, and can represent as much as 4% and 2%, respectively, of the nutrients in the abdominal muscle of juvenile *S*. *verreauxi* [18]. Similarly, the abdominal muscle total lipid content of *J*. *edwardsii* adults is around 4%, 95% of being in the form of phospholipids with up to 30% as essential omega-3 long chain PUFAs [3]. The lack of relationship between abdominal muscle nitrogen content (dry weight) and other nutritional condition indices indicates that the energy released from protein catabolism is via an overall reduction in muscle mass and dry matter content rather than large changes in dry matter composition [18].

Losses in abdominal muscle dry matter and carbon content are of major interest as they ultimately imply lobsters have undergone severe or long-term nutritional stress, whether in the wild or in holding tanks prior to shipment to live markets. Indicators based on abdominal muscle better reflect pronounced starvation and the costly utilisation of tail muscle protein for energy, and are therefore not as affected by the moult cycle position, sex and reproductive status of the lobsters compared to hepatopancreas total lipid [2,11]. This makes them more easily applicable for crustacean with long intermoult cycle where it is difficult to distinguish between lobsters at the beginning, middle or end of the intermoult [6]. The abdominal muscle water content has been proposed as a reliable indicator of condition in *Panulirus longipes* [14], but because it traditionally involves the destruction of the animal, it has remained unsuitable as a field method or for the live lobster market to date.

### Predicting nutritional condition with NIRS

Combining groups of lobsters of different sizes, species and nutritional condition was a productive data management procedure and generated enough variation to encompass the natural range of variation occurring in spiny lobster populations and develop robust universal NIRS models that can be applied to a diversity of samples [31]. The composition of both the abdominal muscle and hepatopancreas was shown to overlap across various lobster species and size-groups which also provided confidence in the development of a generic spiny lobster model for the determination of nutritional condition. The two regions of the spectra used in this study between 9404–7498 cm^-1^ (1063–1334 nm) and 5450–4247 cm^-1^ (1834–2354 nm) relate to important regions for total lipid and moisture, respectively, in meat products [22]. The accuracy of the NIRS models developed from scanning whole lobster tails is comparable to previous NIRS analysis of fish flesh with no sample preparation. Total lipid prediction (R^2^ = 0.90; RMSEP = 1.4%) on whole salmons [21], models developed for dry matter (R^2^ = 0.47–0.79; SECV = 1.79–2.76%) and total lipid (R^2^ = 0.48–0.69; SECV = 2.12–2.81%) on whole sea bass fillets [34], and models for glycogen (R^2^ = 0.77; SECV = 4.24%) in live abalone [24]. In our study, the models’ RPDs ranged between 1.4 and 2.4. Models with RPD> 2 tend to be sufficiently accurate to be considered for screening purposes [21], which was the case in this study for the AM_C_ and HP_TL_ models. An RPD of 2.7 was reported for glycogen models developed from the foot muscle of live abalone [24].

The AM_C_ and HP_TL_ models developed in this study indicate NIRS has great potential as a tool for rapid and non-invasive screening of nutritional condition of live lobsters. For ecologists, fisheries and aquaculture scientists, this technique provides a way to monitor the condition of lobsters under changes in wild and culture conditions, including feed availability, feed quality and water temperature. Peak total lipid values of the hepatopancreas correlate to moult increment and growth in *J*. *lalandii* [2] so this method may provide scope to predict spiny lobster population growth without having to rely on tagging and recapture. For the consumer, low abdominal muscle carbon content is likely to be associated with low meat yield, lower health benefit due to reduced omega-3 lipid, and possibly a reduction in quality attributes such as taste and texture [3]. Grading lobsters non-invasively on this basis may provide an opportunity to closely manage landings or live-holding duration in relation to the lobster capacity to withstand starvation, select poor quality lobsters for processing into frozen or cooked products, and/or attracting price premiums for lobsters with particular meat attributes that satisfy specific market demands [17,35].

The good correlation between AM_C_ and haemolymph TP, and the successful development of a model to predict TP directly in this study (RPD = 1.8), suggests NIRS may find an additional role in predicting lobster vitality for live shipment [17]. NIRS is a more onerous and costly technique than refractometry to predict haemolymph protein, but offers greater throughput capacity, the prediction of other important variables related to product quality, and a non-invasive approach that is less likely to endanger the lobster’ health.

This feasibility study paves the way for the development of spiny lobster condition models using portable non-invasive NIRS devices for use in the laboratory, on boats or at holding facilities. The approach has considerable potential for the management of wild stocks, aquaculture research, the live lobster market, and is potentially transferable to other crustacean species.

## Supporting Information

S1 Dataset Dataset containing all biological and FT-NIR data underlying our findings. (XLSX)
